# Influences of DMP on the Fertilization Process and Subsequent Embryogenesis of Abalone (*Haliotis diversicolor supertexta*) by Gametes Exposure

**DOI:** 10.1371/journal.pone.0025951

**Published:** 2011-10-20

**Authors:** Jin Zhou, Xiao-Shan Zhu, Zhong-Hua Cai

**Affiliations:** Ocean Science and Technology Division, Graduate School at Shenzhen, Tsinghua University, Shenzhen, People's Republic of China; University of Canterbury, New Zealand

## Abstract

Di-methyl phthalate (DMP), a typical endocrine disrupting chemical (EDC), is ubiquitously distributed in aquatic environments; yet studies regarding its impact on gametes and the resulting effects on embryogenesis in marine gastropods are relatively scarce. In this study, the influences of DMP on the gametes and subsequent developmental process of abalone (*Haliotis diversicolor supertexta*, a representative marine benthic gastropod) were assessed. Newborn abalone eggs and sperm were exposed separately to different DMP concentrations (1, 10 or 100 ppb) for 60 min. At the end-point of exposure, the DMP-treated eggs and sperm were collected for analysis of their ultra-structures, ATPase activities and total lipid levels, and the fertilized gametes (embryos) were collected to monitor related reproductive parameters (fertilization rate, abnormal development rate and hatching success rate). Treatment with DMP did not significantly alter the structure or total lipid content of eggs at any of the doses tested. Hatching failures and morphological abnormalities were only observed with the highest dose of DMP (100 ppb). However, DMP exposure did suppress sperm ATPase activities and affect the morphological character of their mitochondria. DMP-treated sperm exhibited dose-dependent decreases in fertilization efficiency, morphogenesis and hatchability. Relatively obvious toxicological effects were observed when both sperm and eggs were exposed to DMP. Furthermore, RT-PCR results indicate that treatment of gametes with DMP changed the expression patterns of physiologically-regulated genes (cyp3a, 17β-HSD-11 and 17β-HSD-12) in subsequent embryogenesis. Taken together, this study proofed that pre-fertilization exposure of abalone eggs, sperm or both to DMP adversely affects the fertilization process and subsequent embryogenesis.

## Introduction

Over the past few decades the world's aquatic environments have been severely impacted by various contaminants from industry, agriculture, urban runoff and other sources. These contaminants can affect all levels of biological organization, from individuals to entire ecosystems [1], [2]. Consequently, decreases in biomass and biodiversity have occurred [3], [4]. Among these contaminants, phthalate esters (PAEs) compose one of the most persistent and widespread groups of xenobiotics in the marine ecosystem [5]. In addition, PAEs are typical endocrine disruptors that can interfere with the hormonal metabolism, immune responses, and reproductive functions of wildlife [6], [7]. The high rates of PAEs production and use have raised concerns regarding its effects on aquatic species. In the last two decades, bio-resources have reportedly declined in both number and quality, affects that could be attributed to adverse effects on the reproduction and development of aquatic animals caused by PAEs contamination [8].

Xenobiotics primarily target marine organisms in their early life stages because these are usually more susceptible to the toxic effects than the adult forms [9], [10]. Thus, great concern has arisen regarding the potential adverse effects of PAEs on the early life stages of marine animals. Early embryos or larvae may be affected by two main pathways: direct exposure or indirect exposure. When directly exposed to phthalate substances, embryos or larvae have demonstrated reduced fertilization rates, impaired embryogenesis and altered larval morphologies [11]–[15]. Indirectly exposure is another pathway by which early embryos or larvae may be damaged. Several studies in aquatic environments have demonstrated the harmful effects of phthalate on embryo survival, growth and morphology by this way in marine organisms, including clam [16], medaka [17] and sea urchin [18].

Direct or indirect exposure of embryos to xenobiotic contaminants (e.g., PAEs) during development clearly causes morphological malformations and developmental dysfunction. However, the question remains as to whether exposure of gametes to these pollutants before fertilization would result in similar developmental defects. Most aquatic organisms release eggs or sperm into their surroundings to fuse and develop as “orphans” in direct contact with the environment. Gametes, directly released into seawater, are unprotected and exposed to environmental contaminants during spawning, so the possibility for contaminants to disrupt the fertilization process and subsequent developmental events is high [19]. Nonetheless, gamete-toxicity has received relatively little attention compared to investigations involving embryos or adults.

Among the PAEs, di-methyl phthalate (DMP) is one the most frequently detected plasticizer and is a relatively stable compound in the natural environment; the hydrolysis half-life is estimated to be about 20 years [20]. In the field of aquatic environments, the reported concentration of DMP ranged from 0.73 to 20 µg/L (Hu et al., 2003) [21]. In this study, the abalone eggs and sperm were exposed separately or in combination to di-methyl phthalate (DMP), to study the effects of this xenobiotic agent on the gametes' fertilization behavior and the subsequent embryogenesis. The questions addressed in this paper are as follows: 1) are the reproductive parameters (fertilization efficiency, morphogenesis, and hatching behavior) adversely affected when gametes are exposed to typical concentrations of DMP found in the environment; and 2) what are the potential mechanisms affecting embryogenesis?

The gastropod (e.g., marine abalone) was chosen as the test species in our study due to its size advantage, environmental sensitivity, short life cycle and breeding characteristics. Most importantly, abalone eggs are semi-translucent and non-sticky, allowing embryonic development to be easily observed using optical microscopy. The species used in this study was *Haliotis diversicolor supertexta*, an important indigenous species in China.

## Results

During the entire experimental period, there were no significant differences between acetone and seawater groups (data not shown). For this reason, the acetone groups were used as controls in the following experiments.

### Effects of DMP on the total lipid levels of eggs and the ATPase activities of sperm

**Figure 1:**
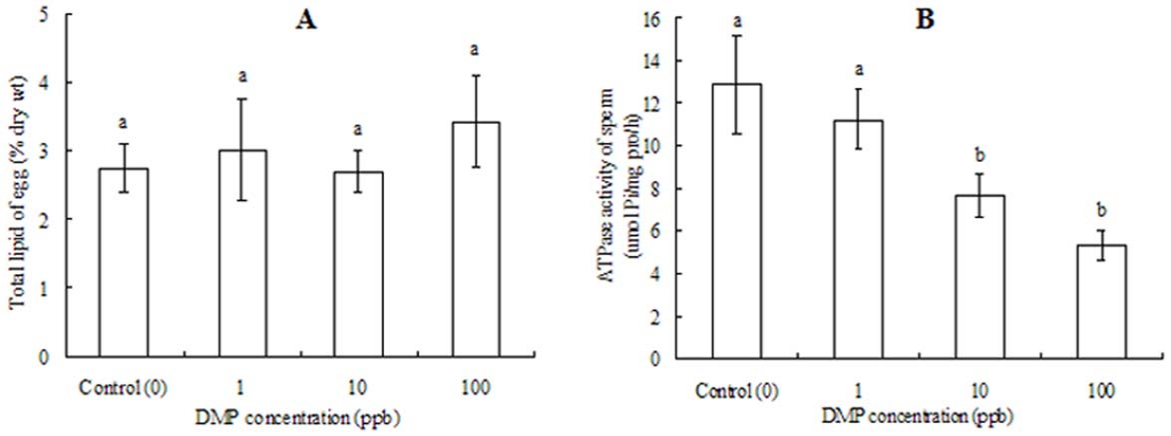
Effects of DMP exposure on total lipid levels of eggs and ATPase activities of sperm. (A) total lipid levels of eggs. (B) ATPase activities of sperm. Each bar represents the mean ± SD. Data are representative of three independent experiments. Significant differences (P<0.05, one-way ANOVA) in total lipid levels and ATPase activities between the experimental and control groups are indicated with different letter.

At the end of the DMP exposure period, the eggs' total lipid content and the sperm's ATPase levels were determined. Total lipid measurements in DMP-treated eggs ranged from 2.69–3.43% (%, dry weight), with no significant differences in total lipid content between treatment and control groups (Figure 1A).

However, spermiotoxicity experiments measured a concentration-dependent decrease in ATPase activities (Figure 1B). ATPase levels significantly declined in higher DMP dose groups, diminishing to 7.65 µmol Pi/mg pro/h at the 10 ppb dose (P<0.05). The lowest ATPase activity (41.4% of the control, P<0.05) was observed when 100 ppb DMP was administered.

### Effects of DMP on gamete morphologies

**Figure 2:**
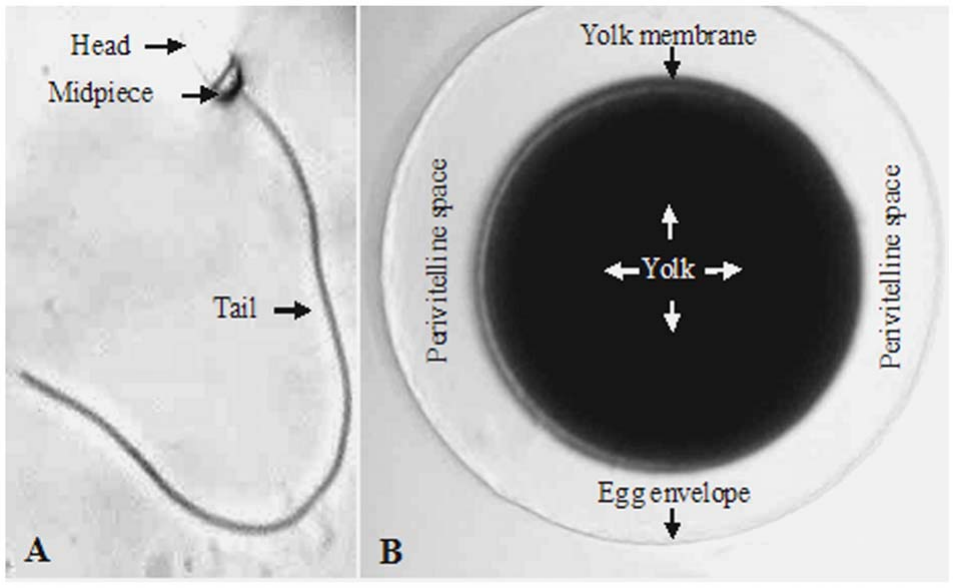
Light microscope images of an abalone sperm (A) and egg (B). The full-length of the sperm is approximately 45 µm, and the diameter of the egg is approximately 150 µm.

The basic morphologies of abalone eggs and sperm are shown in Figure 2. In this study, the basic structures of eggs showed no significant changes between the control and treatment groups (data not shown). Similarly, few apparent structural abnormalities (i.e., teratosperm) were observed in control or DMP-treated sperm.

**Figure 3:**
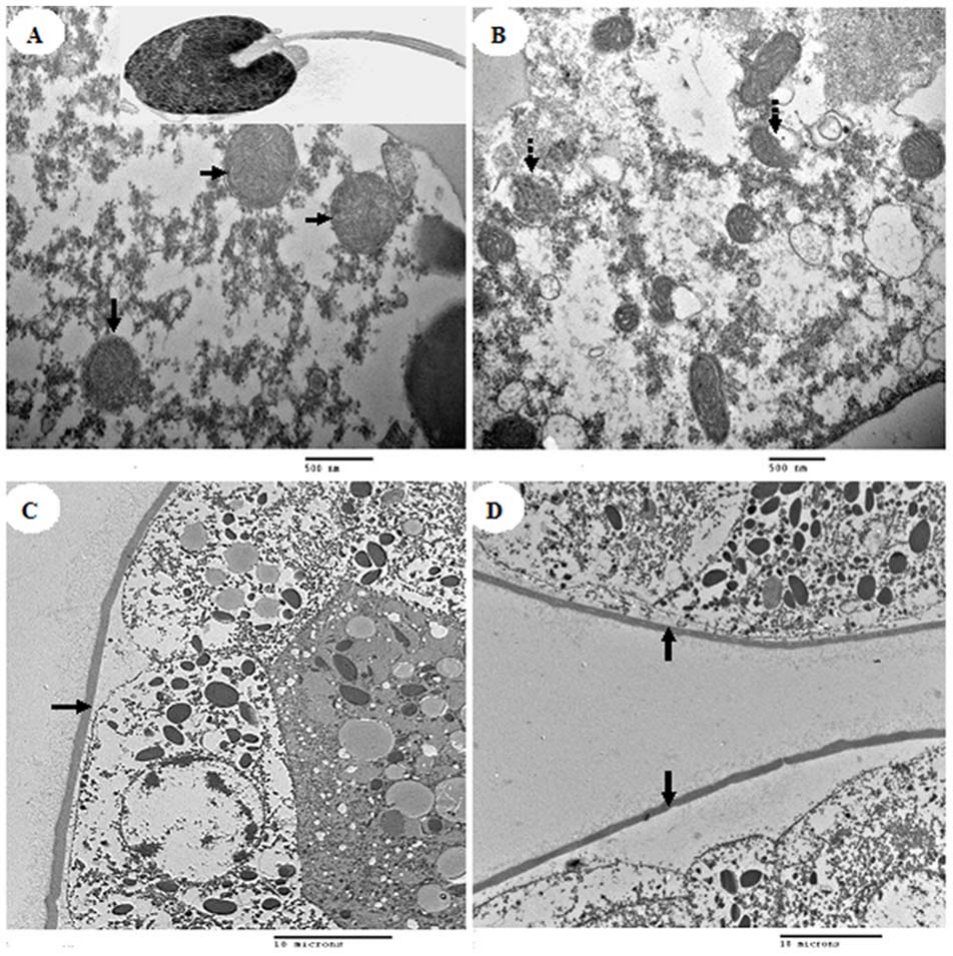
Electron microscopic analysis of the effect of DMP treatment on abalone sperm and eggs. A, the ultrastructure of control sperm (the arrows indicate mitochondria, the insert picture is the full TEM image of the sperm); B, the ultrastructure of DMP-treated sperm (the mitochondria swell and secund cristae are marked by dotted line arrows); C, the ultrastructure of control eggs; and D, the ultrastructure of DMP-treated eggs (arrows denote egg envelopes).

However, TEM analyses of DMP-treated sperm revealed ultrastructural changes. Normal abalone sperm have funnel-shaped heads and whip-like tails (Figure 3A, insert). Under control conditions, the cytoplasm and organelles (e.g., mitochondria) were clearly observed in abalone sperm (Figure 3A). After exposure to DMP for 60 min (see in particular the 100 ppb results), slight swelling or eccentrically placed cristae were observed in sperm mitochondria (Figure 3B).

Likewise, the eggs exhibited relatively few morphological abnormalities in the control or DMP-treated groups. The egg-envelope integrities and internal structures are similar between the control and treated groups (Figure 3 C&D).

### Fertilization rate

**Figure 4:**
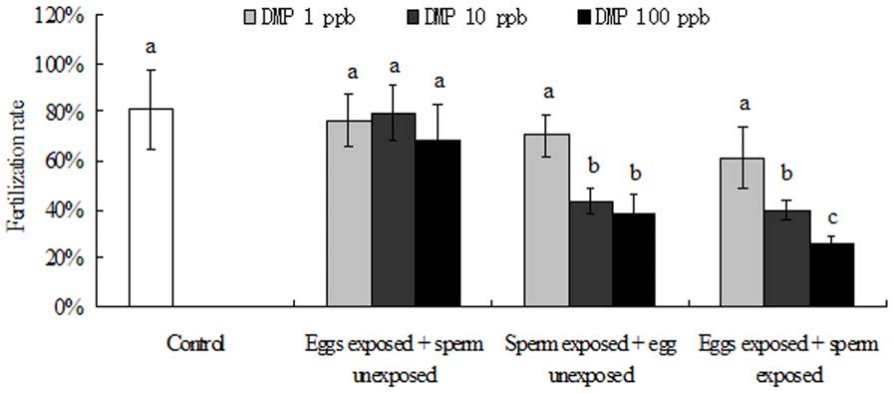
Effects of DMP exposure on fertilization rates (%) of abalone gametes. The percentages of fertilization in different protocols were determined by counting approximately 100–150 randomly sampled eggs. Data are means±SD of three tests. Different letters denote statistically significant differences between control and treatment groups determined by one-way ANOVA (^b^P<0.05, ^c^P<0.01).

The fertilization rates obtained for the T1 samples (eggs exposed to DMP) varied between 68.5% and 79.6%, with no noticeable differences between the control and treatment groups (Figure 4). However, in T2 experiments, the fertilization capabilities were significantly impaired in DMP-treated sperm with the exception of the lowest dose (1 ppb). The percentage fertilization values (%) decreased to 43.6% in the 10 ppb treatment groups and 38.5% in the 100 ppb treatment groups (Figure 4). For the T3 samples (both eggs and sperm exposed to DMP), the fertilization efficiencies declined more dramatically, with lower fertilization rates (approximately 40%) beginning at the 10 ppb dose (P<0.05). The lowest fertilization rate (26.6%) was observed at 100 ppb DMP dose (P<0.01).

### Embryogenesis

#### (1) Abnormal post-fertilization development rates

**Figure 5:**
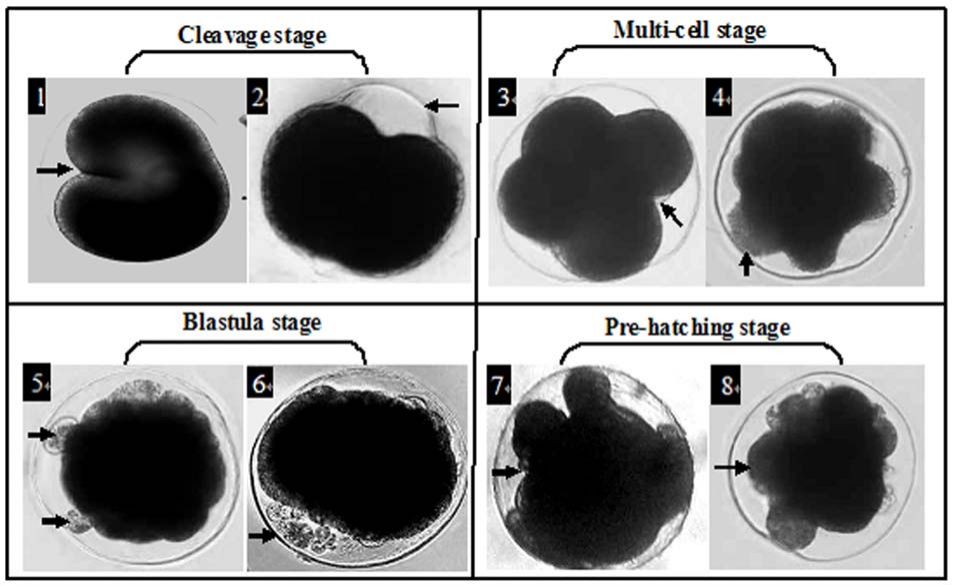
Effect of PAEs on morphological development of abalone embryos. Representative malformations in abalone embryos due to exposure of gametes to DMP (taking sperm exposed to 10 ppb DMP as a example). Arrows point to representative abnormalities for each stage, including incomplete cleavage (1), abnormal protuberances on the embryo envelope (2), dissymmetry splits (3, 4), yolk-sac edema (5, 6) and developmental arrest (7) or monotrochal larva malformation (8).

Post-fertilization development was disrupted to varying degrees when gametes were exposed to DMP. Several gross morphological malformations were observed, including irregular cleavages, asymmetrical splits, yolk-sac edemas, blastula developmental arrests, and monotrochal larva stage developmental malformations. Representative photographs of the abnormal embryos are shown in Figure 5.

**Figure 6:**
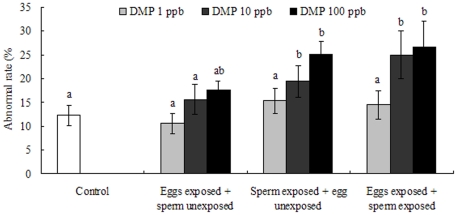
Effects of DMP exposure on embryo abnormality rates (%) of abalone gametes. Values are means±SD, and different letters denote values that are significantly different (P<0.05) and the columns that share the same letter are not significantly different (P>0.05).

After statistical analysis, only eggs exposed to the highest DMP concentration (100 ppb) showed an increase in the percentage of embryos with abnormal development (approximately 17.7%, Figure 6). In contrast, sperm displayed more susceptibility to DMP poisoning. At 8 hpf, solvent control experiments resulted in an embryo abnormality rate of approximately 12.3%, while 1, 10 and 100 ppb DMP treatment groups showed 15.3%, 19.5% and 25.3% embryo abnormality rates, respectively. The abnormality rates increased in a concentration-dependent manner. Similar results were observed when both eggs and sperm were exposed to DMP, revealing a strong positive correlation between the percentage of abnormally developing embryos and the treatment dose (Figure 6). At the highest dose, 26.7% of embryos exhibited abnormal development and the values was significant difference compared with those of the control group (P<0.01).

#### (2) Hatch success rates of post-fertilization development

**Figure 7:**
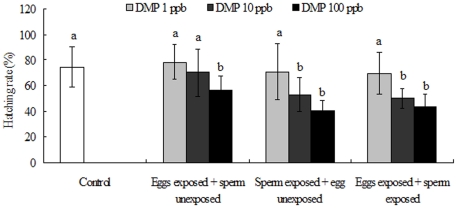
Effects of DMP exposure on Embryo hatching rates (%) of abalone gametes. Values are means±SD, and different letters denote values that are significantly different (P<0.05) and the columns that share the same letter are not significantly different (P>0.05).

Hatch efficiencies were measured after exposure of eggs, sperm or both to DMP. In T_1_ samples (eggs exposed to DMP), compared with the controls, hatch success rates were not significantly different from controls when eggs were exposed to low (1 ppb) or medium (10 ppb) DMP concentrations. However, a high dose of DMP (100 ppb) resulted in a lower number of hatching-out embryos than observed under control conditions (P<0.05, Figure 7). Spermiotoxicity experiments (T_2_ groups) demonstrated a concentration-dependent decrease in hatch rate. Under control conditions, the hatch success rate was approximately 75.0%, with little difference in the percentage of hatching-out embryos compared to 1 ppb DMP conditions. However, the number of hatching-out embryos was dramatically lower in the 10 and 100 ppb DMP treatment groups (a 1.41-fold and 1.85-fold decrease, respectively) (P<0.05). A similar trend was also observed for the T3 groups with fewer embryos hatching-out successfully at higher DMP doses. In the 100 ppb groups, more than half the embryos lost their hatching-out competence (Figure 7).

### DMP exposure leads to alteration in hormonal-related genes expression

**Figure 8:**
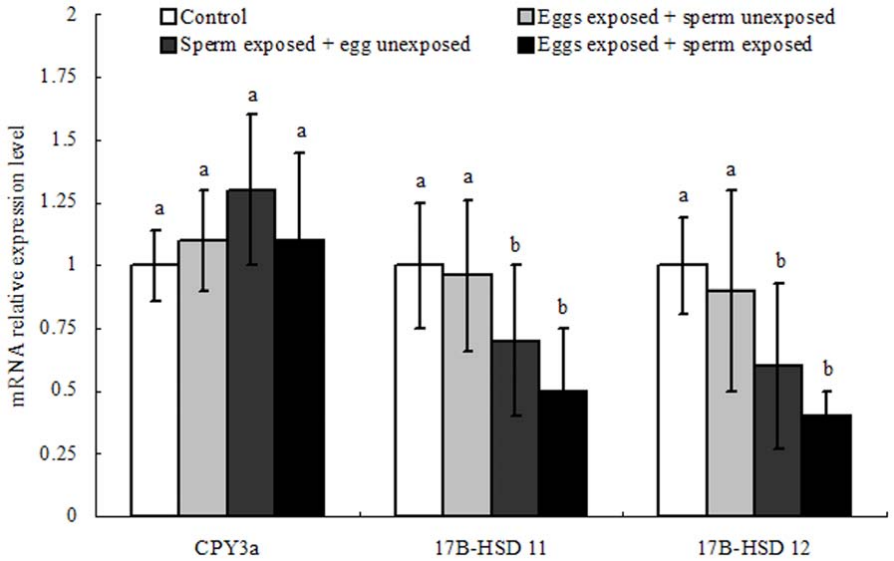
Effect of DMP on reference genes expression in abalone embryos. Expression levels of cyp3a, 17β-HSD-11 and 17β-HSD-12 after exposure of sperm, eggs or both to DMP. Each bar represents the mean±SD. All real-time reactions were performed three times. Significant differences among the groups were indicated with different letter.

To ascertain whether DMP-induced embryotoxicity was accompanied by stress dysfunction and physiological dysregulation, the genes (cyp3a, 17β-HSD-11 and 17β-HSD-12), implicated in abalone embryo detoxification and steroid regulation were measured [15], [22]. As shown in Figure 8, embryo 17β-HSD-11 and 17β-HSD-12 mRNA levels were mildly suppressed in DMP-treated eggs compared to control. Exposure of abalone eggs to DMP did not result in significant changes in cyp3a during the post-fertilization development period. A more obvious homeostasis disruption of post-fertilization development was exhibited in spermiotoxicity experiments. In these experiments, in which DMP-exposed sperm fertilized unexposed eggs, the embryo mRNA levels of 17β-HSD-11 and 17β-HSD-12 decreased significantly (1.43- and 1.67-fold, respectively) compared to control levels. The similar profiles were observed when both gametes were exposed to DMP before fertilization. In these experiments, a more obvious result was observed for these genes, and mRNA levels were altered more significantly compared to results obtained from experiments in which only one gamete was exposed to DMP (Figure 8).

## Discussion

Toxicological parent to progeny developmental effects, particularly non-genetic contributions to offspring ontogenesis, has received much attention in past several decades [23]. In this regard, the maternal or paternal effects of xenobiotics in aquatic animals have been widely investigated. The results have demonstrated that exposure of the mother or father to contaminants at environmentally relevant levels can cause gonad growth retardation, fertilization dysfunction, embryonic development defects, and transmissible damages to the offspring [24]–[30]. However, studies on gametes are relatively scarce. Because external fertilizers (e.g., broadcast spawners) release their gametes freely into the environment, these organisms should be more vulnerable to fertilization failure as a result of point-of-contact chemical exposure. Thus, it is important to understand the effects of xenobiotics on gametes and subsequent reproductive processes. The present study investigates the toxicity effects of DMP on germ cells. To our knowledge, this study is the first to expose abalone gametes to DMP in order to assess the effects on the fertilization process and subsequent embryo development.

The present study reveals no significant adverse effects on fertilization efficiency following exposure of eggs to DMP. This finding is consistent with earlier studies on other marine invertebrates, in which acute exposure of eggs to contaminants had no effect on fertilization abilities [31], [32]. The eggs showed no significant differences between the experimental and control groups with regard to ultrastructure and total lipid levels (Figure 1A; Figure 3 C&D), indicating that DMP exposure did not obviously affect the fundamental structure of the egg-envelope or the lipid content. From these results, we speculate that, at least in the case of short-term or low-dose exposure, egg exposure to DMP alone cannot account for the fertilization defects observed in the wild [33]. The slight decline in fertilization rates observed in the highest-dose group is possibly due to the accumulation of high DMP concentrations in the egg envelope surfaces and micropyle blockage affecting sperm/egg fusion [34]. However, further research is needed to evaluate the barrier actions of DMP on egg envelope micropores.

Sperm were more sensitive than eggs to DMP exposure. DMP exerted a potent concentration-dependent inhibitory effect on sperm fertilization ability (Figure 4). These toxicological results indicate that short-term exposure to DMP may reduce sperm function. Previous literatures have also reported the influences of xenobiotic pollutants on sperm functions and fertilization processes. The related mechanisms include reducing sperm swimming speed [33], damaging sperm DNA structure [8], disrupting flagellum Ca^2+^ signaling [19] and increasing the probability of aneuploid sperm [35]. In the present study, we observed the declines of ATPase activity on sperm, which indicate that DMP-treated sperm have lower energy at the time of fertilization than control sperm. As ATPase is responsible for releasing ATP (the enzymatic contents), and ATP aids sperm penetrating into the eggs [36], suppressed ATPase activity may decrease the energy for propelling the sperm to the egg, which eventually results in a diminution of the fertilization ratio.

The impaired fertilization ability of DMP-treated abalone sperm may also be explained by associated ultrastructural changes. As shown in Figure 3B, DMP caused slight mitochondrial swelling and distraction of cristae in sperm midpieces. The deformation of mitochondria may be attributed to DMP interaction with mitochondrial membrane proteins, resulting in increased membrane permeability to cations and anions [37]. Evidence suggests that membrane damage may correlate with altered sperm function and decrease fertility [38], [39]. Taken together, these results suggest that DMP exposure influences the sperm energy status and ultrastructure, providing possible mechanisms leading to the fertilization impairment of abalone gametes during the early phase of the fertilization process.

In addition to impacting the fertilization process, acute exposure of gametes to DMP disrupted abalone embryogenesis. In T1 test groups (exposure of eggs), the development process was not significantly affected by low or medium concentrations of DMP. One possible reason for this result is that the egg envelope acts as a protective layer, resisting the intrusion of DMP. Nevertheless, a hatching-out delay appeared at the highest dose of DMP. High concentrations of xenobiotic may interfere with permeability of the outer membrane of egg cells. Under these circumstances, fertilized eggs would suffer metabolic deactivation, ultimately affecting the morphogenesis or hatching behavior [40]. Significant increases in the frequencies of developmental abnormalities and hatch defects were observed in T2 compared to T1 experiments. Because they exhibit a limited capacity for DNA repair and antioxidant defense [41], sperm are potentially more susceptible to DMP-induced damage than oocytes. In addition, we speculated that after DMP exposure, the foreign xenobiotic substance may accumulate in the sperm body, making the sperm cell act as a toxicity vector that transfers DMP into eggs, thereby forming DMP-contaminated embryos (fertilized eggs) and influenced embryonic development process. Experiments designed to detect DMP levels in sperm cells are presently underway in our laboratory to further test this hypothesis.

During the fertilization process, each gamete provides components and signals that are essential to normal embryonic development. In many invertebrates, eggs have large stores of maternal mRNA and proteins synthesized before fertilization [42], while the sperm supplies factors that initiate the developmental program. Exposure of gametes to environmental toxicants may lead to alteration of these essential components, causing fertilization failure and/or defective development [33]. Among these components, the cytochrome P450 (CYP450) isoenzyme is particularly important. In early stages, CYP450 is responsible for the degradation of contaminants and affects cell differentiation and ontogenesis [43]. Previous studies report cyp3a over-expression in zebrafish and medaka embryos after exposure to TBT or TPT [44]. However, the present results show no significant difference in cyp3a expression between experimental and control groups, perhaps due to the use of different exposure substances and durations.

In vertebrate, sex steroid hormones play an important role in early embryo development, including regulating differentiation, development and homeostasis [45]. Steroid hormones are synthesized from cholesterol through the action of steroidgenic enzymes, which are regulated primarily at the transcriptional level [46]. The enzymes 17β-hydroxysteroid dehydrogenases (17β-HSDs) play a key role in the cascade of steroidogenesis and the activy of these enzymes used as indicator of steroid biosynthesis [47]. Previous studies have shown that these enzymes are sensitive to adverse environmental conditions during embryogenesis in various vertebrate animals, including rats [48], frog [49], and fish [44]. In this study, the abnormal expression of types 11 and 12 17β-HSD mRNA was observed after abalone (an invertebrate species) gamete exposed by DMP. Despite the lack of data on steroids receptors and functional studies during embryogenesis in invertebrate, we may also hypothesize a similar role of 17β-HSD during mollusks embryonic development process. We cannot totally rule out a role of 17β-HSD in regulating steroid biosynthesis during abalone embryo development. In virtually, the biological function of 17β-HSD-11 [submitted manuscript] and 17β-HSD-12 [50] were verified by transiently transfecting their ORF in HEK-293 cells, which provided further support that abalone 17β-HSD may participate steroid hormone metabolism. Besides, the physiological function of 17β-HSD involved in steroidogenesis or the cascade process has also been demonstrated in sea urchin [51], mussel [52], and dogwhelk [53]. Taken together, 17β-HSD may be a biomarker, at least in part, for abalone embryo developmental impairment and hatching dysfunction. However, this cannot be extended to all mollusks as 17β-HSD receptors and binding competence are not yet fully understood. Further investigations including mRNA expression and protein activity measurements are thus required to conclude on the exact physiological role(s) of these 17β hydroxysteroid dehydrogenase enzymes in steroidogenesis function during mollusks embryogenesis.

### Conclusions

Clearly acute DMP exposure of abalone eggs or sperm adversely affects fertilization efficiency and subsequent embryogenesis. Sperm were more sensitive to DMP than eggs. The results suggest that compromised sperm energy status contribute to fertilization defects in germ cells. Disruption of the physiological performance of abalone embryos is a possible mechanism leading to phenotypic changes of embryogenesis, such as developmental abnormalities and decreased hatchability. In the next step, multiple hormone-regulated genes and their interactive, simultaneous effects on morphology, physiology, and development will be examined to better understand the mechanisms of early-life-stage toxicity of plastic contaminants.

## Materials and Methods

### Chemicals and reagents

DMP was obtained from Shenggong, Co., Ltd. (Shanghai, China, purity ≥97%). Stock solutions were prepared in acetone (Dingguo, Beijing, China), which was also used as the vehicle control. The final acetone concentrations in each treatment were the same (0.005%). Trizol, PCR reagents and supplements were obtained from Sigma (Beijing, China), Invitrogen (USA) or TaKaRa Co., Ltd. (Dalian, China). All other reagents were of analytical grade.

### Animals and ethics statement

**Figure 9:**
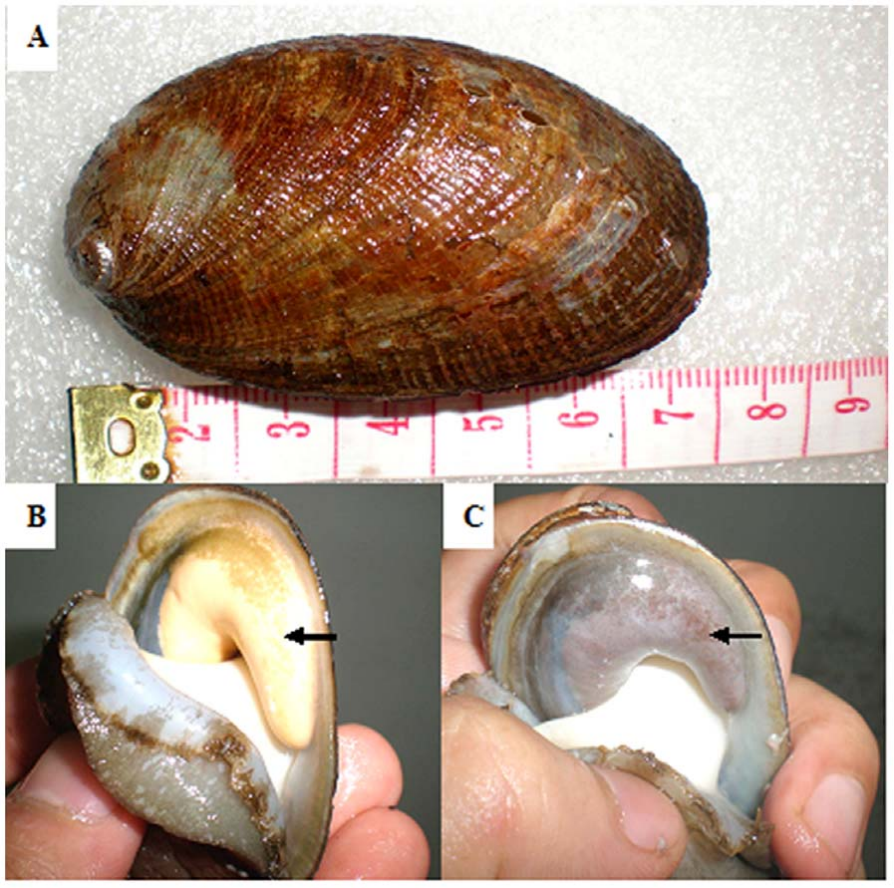
The experimental animals in this study. A, the fully-mature abalone (shell-length near 7 cm); B, the male individual (note the yellowish-white spermary); and C, the female individual (note the dark-violet ovary).

The abalones (two years old, average shell length 7.0±0.8 cm) were obtained from a local hatchery, Kinghaili Aquaculture Co., Ltd (Longgan District, Shenzhen city, China). Abalone maturation was confirmed by gonad inspection. Fully mature males exhibited yellowish-white spermaries, whereas mature females had dark-violet ovaries (Figure 9). Gonad-outstanding individuals were selected and used in this study.

All experiments were performed in compliance with the Chinese animal protection law. The care and use of laboratory animals was approved and supervised by the Tsinghua University Animal Care and Use Committee, complying with the rules of Regulations for the Administration of Affairs Concerning Experimental Animals (Tsinghua University local representative of the animal welfare agency, approved by the State Council of P.R. China). The individual experiments are approved by Tsinghua University Home office inspectors (Home Office Project licenses: CGSZ10-4755).

### Gametes' preparation

Female and male abalones were kept in separate rectangular tanks containing aerated sand-filtered seawater (SFS), in which they were held for up to 24 h prior to experimental treatment. Culture conditions were as follows: water temperature 24±2°C, salinity 30±2‰, pH 8.0±0.2, and dissolved oxygen no less than 6.0 mg/L. Abalones (♂∶♀≈1∶3) were induced to spawn in ultraviolet-radiated seawater [54]. Before spawning, the animals were washed several times with SFS. The newborn sperm were placed in a sperm/water mixture in a clean container (0.5 L). The newborn eggs were filtered through a 100 µm mesh nylon net, gently washed two times with SFS and re-suspended as an eggs/water mixture in 0.5 L clean seawater. The gametes (eggs and sperm) were then used for the following experiments. Gametes production, artificial fertilization and embryo maintenance followed the Yang and Yan method (2006) [55].

### Exposure experiments

The following three experimental designs (trials) were carried out in this study: eggs exposed to DMP but sperm unexposed (T1); sperm exposed but eggs unexposed (T2), and both sperm and eggs exposed (T3). The abalone eggs in the T1 group were exposed to DMP at three different concentrations (1, 10 or 100 ppb) for 60 min, followed by fertilization with unexposed sperm. The abalone sperm in the T2 group were exposed to DMP at the same doses for 60 minutes and then used to fertilize unexposed eggs. In the T3 group, both sperm and eggs were exposed to DMP using the same protocol and were then used to fertilize each other. In all experimental groups, the fertilized eggs (embryos) were developed in clean seawater. The same volume of acetone solvent was used as the control, and SFS was used as the blank. Three replicates were conducted for all treatments and controls.

### Total lipid levels of eggs and ATPase activities of sperm

Samples of exposed eggs (after 60 min) were collected and used for total lipid content analyses performed according to the methods of Floch et al. (1957) [56].

Sperm samples for ATPase enzyme assays were obtained by homogenizing the sperm cells as described by Levine et al. (1978) [57]. ATPase activities were measured using the ATPase colorimetric assay kit based on its instructions (Jiancheng Co., Ltd., Nanjing, China). Briefly, 20 µl samples were added to 250 µl ATPase reaction mixtures and incubated for 30 min at 37°C. Rates of ATP hydrolysis were determined to be linear over this period of time. Unless otherwise indicated, all ATPase assays were performed in this manner. For each measurement, the extent of ATP hydrolysis was determined by measuring the amount of inorganic phosphate using the colorimetric procedure. The protein concentration was determined using the Bradford (1976) method [58]. One unit of enzyme activity is defined as the amount of enzyme resulting in a hydrolysis rate equal to 1 µmol Pi/mg pro/h.

### Morphology analysis of sperm and eggs

The basic sperm morphologies were evaluated by light microscopy (Nikon, Japan). Sperm were fixed with 2.5% buffered glutaraldehyde. Approximately 200–300 sperm per sample were analyzed. Basic morphology analyses focused on detecting whether the existence or absence of teratosperm (e.g., angulated sperm or sperm suffering breakage). In addition to basic morphology analyses, sperm from the control groups and the DMP-treated groups were processed utilizing transmission electron microscopic (TEM) analysis. Briefly, samples were fixed with 2.5% glutaraldehyde in 0.1 M phosphate buffer, then washed in 0.1 M cacodylate buffer and postfixed with 1% osmium tetroxide. Subsequently, the samples were washed with buffer, dehydrated, and embedded in Epon 812. Ultrathin sections (70 to 90 nm thick) were obtained using an ultramicrotome (Lecia EM UC6, Germany) and were mounted on 150-mesh copper grids. Imaging was performed with an H-7650 transmission electron microscope (HITACHI, Japan) and a Gatan 832 CCD camera.

Egg morphologies were detected utilizing similar methods.

### Fertilization rate

The aim of this set of experiments was to assess the fertilizing ability of gametes that had been exposed to varying DMP concentrations, not to assess how fertilization proceeds in DMP-polluted water. Therefore, prior to fertilization trials, aqueous DMP in the sperm/water (or eggs/water) mixtures required removal. Accordingly, in T1 samples, the DMP-treated eggs were filtered through a 100-µm-mesh nylon net, then gently washed two times with filtered seawater before being fertilized with non-DMP-treated sperm. In T2 samples, the DMP-treated sperm were placed in 1.5 mL Eppendorf tubes and centrifuged at low-speed for a few minutes, forming a pellet of sperm at the bottom of the tube. A 900 µl aliquot of the supernatant was decanted and replaced with filtered seawater. The resulting sperm/water solution was then used to fertilize non-DMP-treated eggs. Centrifuging the sperm/water solution did not adversely affect sperm viability, as demonstrated by a preliminary study in which centrifuged sperm exhibited motility comparable to non-centrifuged sperm and were able to successfully fertilize eggs [59]. The same protocol was used for T3 samples.

Fertilization trials were conducted by mixing sperm with eggs *in vitro*. Sperm and eggs were allowed to interact in an aerated vial for 30 min, at which time 0.5 mL of buffered glutaraldehyde was added to arrest fertilization and preserve developing fertilized eggs (embryos). Fertilization rates were determined by counting the ratio of fertilized/total eggs from a randomly selected sub-sample of at least 100 eggs (range 100–150). Fertilized eggs exhibiting characteristic polar bodies are clearly visible under 200×magnification.

### Development parameters in embryogenesis

Abnormality rate (%). For these experiments, a multi-cell stage was adopted to determine the cleavage blocks. The blastula stage (3 hours post fertilization (hpf)) was the starting point for detecting abnormalities, and the monotrochal larva stage (8 hpf, ready to hatch) was the end point for abnormal rates determination. Embryo samples were collected randomly, fixed with formalin, and then observed under a light microscope (Nikon, Japan). Each treatment was repeated three times. For each treatment, 300 embryos (100×3 replicates) were used to measure the abnormality rate, which was calculated according to the formula below:
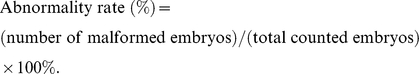



Hatching success rate (%). Each hatching rate was calculated at the end of the hatching-out stage. After the monotrochal larva stage (about 9 hpf), the embryo hatches and becomes a swimming veliger larva. Embryos that exhibited developmental arrest, hatch delays or did not hatch were recorded as hatching failures. For each treatment, 300 embryos (100×3 replicates) were chosen to measure the hatching success rate, which was calculated as follows:
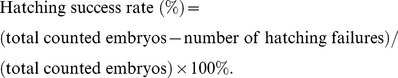



### Real-time quantitative PCR

At 8 hpf, samples from each treatment group were collected for reference genes' expression analysis. Three physiologically-related genes, cyp3a, 17β-HSD-11 and 17β-HSD-12, were selected to assess the effects of DMP on the development of abalone embryos. The completed cDNA sequences of cyp3a, 17β-HSD-11 and 17β-HSD-12 were obtained by homology cloning in our previous work. The full-length cDNA sequence was submitted to GenBank, and the accession numbers were GU984784, HQ699520 and GU984783, respectively. The gene-specific primers for cyp3a, 17β-HSD-11, 17β-HSD-12 and the internal control β-actin (accession number AAS00498) were used to amplify amplicons specific for *H. diversicolor supertexta*. The sequences of primers and the length of amplicons were shown in table 1. The fluorescent real-time quantitative PCR amplifications were performed according to previously described methods [15]. Amplification products were subjected to melting curve analysis to confirm that a single PCR product had been amplified and detected. Each sample was analyzed three times to confirm reproducibility. β-actin transcript levels were used to normalize the results, and negative control reactions, in which template cDNA was omitted, were included for every primer set. Relative amounts of target genes and β-actin RNA in all cDNAs were calculated from a standard curve. To maintain consistency, the baseline was determined automatically using SDS software (V.1.3.0, ABI, USA). Target mRNA levels were expressed in relative levels (target mRNA/β-actin-RNA).

**Table 1:** Primer sequences for real-time quantitative RT-PCR analyses.

Target Gene	GenBank Accession Numbers	Primer Sequence (5′-3′)	Product (bp)
*Cyp3a* (Fr)	GU984784	TCCCAAACTGGAGAAAGG	226
*yp3a* (Rr)		AGAGTCGTAACGGTGCT	
17β-HS-11 (Fr)	HQ699520	CCACCCGCTAGACAAGAT	170
17β-HS-11 (Rr)		CCAATACGACGGACCTCA	
17β-HS-12 (Fr)	GU984783	TGACCAGCATTGTGACGC	289
17β-HS-12 (Rr)		CCCAGTGTAGCAACAGCC	
β-actin	AAS00498	GTCTTTCCCTCCATCGTCGGAC GTCCCAGTTGGTGACGATTCCG	260

CYP3a: Cytochrome P450, family 3, subfamily A;

17β-HSD-11: 17β-hydroxysteroid dehydrogenases 11;

17β-HSD-12: 17β-hydroxysteroid dehydrogenases 12.

### Statistical analyses

Data related to developmental parameters and physiological features were analyzed by one-way analysis of variance (ANOVA) accompanied by Tukey's multiple comparison analysis using SPSS 11.0 software (USA). The qRT-PCR data analyses were executed by comparative CT method (2^−ΔΔCT^ method) using SDS software V1.3.0 (ABI, USA) [60]. All values are presented as mean ± SD, with P<0.05 considered statistically significant.
